# Changes in Correlates of Health-Related Quality of Life Between Children with Spina Bifida and Their Parents as Influenced by Their Level of Independence in Toileting Self-Management: A Cross-Sectional Study

**DOI:** 10.7759/cureus.60526

**Published:** 2024-05-17

**Authors:** Tae Kawahara, Akemi Yamazaki

**Affiliations:** 1 Pediatric and Family Nursing, Division of Health Sciences, Graduate School of Medicine, Osaka University, Suita, JPN

**Keywords:** health-related quality of life, transition, spinal dysraphism, parent, incontinence, child

## Abstract

Background

Spina bifida (SB) leads to various complications, such as bladder and bowel disorders, which can significantly impact quality of life (QOL). Parents of children with SB are often heavily involved in bladder and bowel management, which can affect their own QOL. Therefore, transitioning to independent bladder and bowel management is pivotal because it influences the QOL of both children with SB and their parents. In this study, we investigated changes in health-related quality of life (HRQOL) among children with SB and their parents in the process of attaining independence in bladder and bowel self-management.

Methods

Children with SB aged 8-17 years and their parents completed the Japanese version of the QOL assessment in SB for children/teenagers (QUALAS-C/T-J) and the Short Form-8 (SF-8). Independence in bladder and bowel management was assessed using a visual analogue scale (VAS). We calculated the correlation between children’s or parents’ HRQOL and the children’s level of independence in bladder and bowel management. Additionally, we conducted a Mann-Whitney U test on the scores of the higher and lower independence groups. The correlation between parent and child HRQOL was analyzed by dividing children’s independence into two groups.

Results

This study consisted of 83 parent-child pairs. Parents’ and children’s HRQOL and levels of self-management independence were not significantly correlated, either overall or by level of independence. The parent-child group with less independence, especially in bowel management, showed moderate to strong HRQOL correlations, whereas the group with more independence showed weaker correlations.

Conclusions

The strength of the correlation for parent-child HRQOL was found to change based on the level of independence in bladder and bowel self-management. These results suggest that the strength of parent-child cohesion tends to be pronounced in regard to the children’s degree of independence in bowel management.

## Introduction

Spina bifida (SB) is a malformation of the neural tube that does not close during fetal development, which results in a variety of complications, including hydrocephalus, bladder and bowel disorders, and lower limb sensory dysfunction. In particular, bladder and bowel disorders are managed at home throughout the patient’s life, often using clean intermittent catheterization (CIC) for urinary management and various methods of bowel management, such as enemas, stool extraction, and progressive enemas, depending on individual requirements [1].

It has become common for children with SB to enter regular classes if they can manage their bladder and bowel problems appropriately in their daily lives. For children with SB with disabilities, it is important to assess how they perceive living with a disability, rather than just the disability itself. Quality of life (QOL) is a concept that can be used as an indicator to assess the perception of one’s daily living. According to the World Health Organization, QOL is defined as an individual’s “perception of their position in life in the context of the culture and value systems in which they live and in relation to their goals, expectations, standards and concerns”, with subcategories of general QOL and perceptions of the influence and impact of health status on QOL (i.e., health-related quality of life (HRQOL)) [2]. For children with SB, it is appropriate to consider the concept of HRQOL, which includes the impact of bladder and bowel problems on their daily lives [3]. Previous studies related to the HRQOL of children with SB have mostly shown that they have lower HRQOL compared with healthy children [4,5]. On the other hand, Olesen et al. reported that children with SB showed higher HRQOL than healthy children in relation to their parents and other supportive factors [6]. In particular, they tended to hide their bladder and bowel disorders more than their physical disabilities, and their bladder and bowel disorders were strongly associated with and an important determinant factor of their HRQOL [7-9]. However, many of these studies were conducted using instruments that measure HRQOL by function (i.e., what the child can do) rather than their perception of living with SB, and therefore, may give a false picture of HRQOL in this population [3]. For example, using a functional question such as “Have you been able to run well?” would inevitably result in a lower score for children with SB with limited walking ability. On the other hand, a subjective question such as “Do you want to run?” reveals what they value in life, and this question is more in line with the definition of QOL proposed by the World Health Organization [2]. The HRQOL of school-age children and adolescents and young adults with SB has been shown to be affected by the relationship between them and their parents and families [6]. Children with SB tend to depend on their parents, and many parents tend to be overprotective and over-involved in their children’s lives [10].

The parents who work with their children with SB to manage bladder and bowel care have been found to structure their lives around bladder and bowel management for everyday management and unexpected incontinence [11]. They tend to cooperate to hide bladder and bowel disorders from people around the children with SB [7]. Parent HRQOL is affected by the stress of SB, parent-child communication, and an increased tendency to have depressive symptoms compared with parents of healthy children [12-13]. On the other hand, improvements in the bowel conditions of children with SB lead to decreased degrees of anxiety and depression among parents and concern regarding bowel incontinence among parents and children with SB [14]. Parents of children with SB are concerned that their children need parental help to complete and may not be able to manage complex self-management, such as bowel control, on their own [15]. Parents are also more likely to direct children’s self-care, especially during puberty, and parental behavior and psychological control over children with SB affect children’s adherence to medical care [10,16]. It has also been reported that parent-child cohesiveness changes in the process of transitioning to bladder and bowel self-management [17]. These findings suggest that appropriate bladder and bowel management for children with SB has a strong impact on the HRQOL of not only children, but also their parents. The cohesiveness of parents and children might become further strengthened during the transitional phase, during which time, the quality of self-care is variable and a common problem for each of them in their daily lives, which might have a strong impact on both the parents’ and children’s HRQOL in relation to bladder and bowel self-management. Given this background, in the present study, we aimed to investigate the effect of the level of independence in bladder and bowel management on parents’ and children’s HRQOL, and whether the correlation between parent-child HRQOL is enhanced in transitional situations with lower levels of bladder and bowel self-management.

Hypotheses and definitions in this study

We examined the following four hypotheses to achieve our aims:

Hypothesis 1. Does the HRQOL of children with SB differ between those who have a low or high level of independence in bladder and bowel management?

Hypothesis 2. Does the HRQOL of parents of children with SB differ between those whose child has a low or high level of independence in bladder and bowel management?

Hypothesis 3. Is there any correlation between the HRQOL of children with SB and their parents?

Hypothesis 4. Is there a stronger correlation between the HRQOL of children with SB and their parents if the children have less independence in bladder and bowel self-management?

In this study, “independence” was defined as children with SB controlling their own bladder and/or bowel management while using the environment and others as tools [17].

## Materials and methods

Ethical considerations

The study protocol was approved by the Institutional Review Board of Osaka University (approval No. 18473, February 15, 2019). The work described was carried out in accordance with The Code of Ethics of the World Medical Association (Declaration of Helsinki). All parents provided written, informed consent before the study began. The study was explained to the children and adolescents with SB, and whether they understood the requirements and agreed to participate was verified. If a child showed comprehension of the explanations provided, verbal and written consent for participation was obtained.

Data collection

The sample size was calculated using a significance level of 0.05 and a power of 0.80. The sample size was calculated using G*power with an effect size (r) of 0.3 (moderate), which required a minimum of 82 pairs of children with SB and their parents to participate [18]. The survey was conducted for four months, from December 2019 to March 2020. The study invitation was mailed to parents of patients of the Japan Spina Bifida Society’s target age, and questionnaires were sent by mail to a total of 96 pairs of parents who had indicated their agreement to participate and participated in previous surveys. The inclusion/exclusion criteria were confirmed by surgical age on the return form, age on the questionnaire, and response status on the Japanese version of the quality of life assessment in spina bifida for children (QUALAS-C-J) and for teenagers (QUALAS-T-J). To help ensure that children with SB and their parents responded to the questionnaires independently, we instructed them to respond in separate rooms, or that the parents assist their children in responding only after completing the survey themselves.

Inclusion and exclusion criteria

In this study, the survey included children with SB aged 8-17 years and their parents. The exclusion criteria were children without a definite diagnosis of SB, who were unable to respond to self-administered questions or interviews, who had not had spinal repair surgery within the first year after birth, and who had no problems with both bladder and bowel function.

Instruments

Demographic Attributes of Children with SB 

The demographic characteristics of the children (age, sex, type of school, ability to walk, presence or absence of shunts, CIC, social bladder continence (dry period ≥ 4 h), degree of incontinence, urinary stoma, bladder enlargement, and main person involved in bladder and bowel management) and parents (relationship, age, marital status, family members living together, presence of child care partners, satisfaction with child care cooperation, employment status, household income satisfaction, education history, and psychiatric history attributes) were reported by the parents. The reliability of the children’s self-administered responses was checked for cognitive developmental level by school type, and any discrepancy in responses to a particular question (e.g., whether the child was managing their own urination) was considered deficient. The representativeness of the population was also confirmed by the rate of CIC implementation.

HRQOL of children with SB

The QUALAS-C and QUALAS-T are self-reported HRQOL instruments for children with SB that were developed by Szymanski et al. [19,20]. The QUALAS-C/T both consist of 10 items and the following two domains: “Esteem and Independence” or “Family and Independence”, and “Bladder and Bowel”. The authors developed the Japanese version of the QUALAS-C (QUALAS-C-J) and QUALAS-T (QUALAS-T-J) for children with SB living in Japan, and the reliability and validity for Japanese children with SB have been verified [21,22]. These instruments are scored on a five-point Likert scale. The total score for each domain ranges from 0 to 100, with higher scores indicating higher HRQOL for children with SB. These instruments contain unique age-specific items addressing independence [8,19]. In the interpretation of HRQOL, the degree of independence in bladder and bowel management is used as the principle parameter instead of age. For ease of interpretation, the Esteem and Independence and Family and Independence domains on the QUALAS-C/T were merged into a Psychosocial domain.

Parents’ HRQOL

The Short Form (SF) series has been widely used as a parental HRQOL instrument in previous studies related to parent and child HRQOL [23]. The Short Form-8 (SF-8) is an eight-item scale that is a shortened measure of the MOS 36-Item Short Form Health Survey (SF-36) [24]. The SF-8 consists of the following domains: General Health, Physical Functioning, Role Physical, Body Pain, Vitality, Social Functioning, Role Emotional, and Mental Health. The SF-8 is an HRQOL scale for people aged ≥ 16 years, and each domain score is calculated along with physical component summary (PCS) and mental component summary (MCS) scores. Higher scores indicate higher HRQOL. Permission to use the SF-8 questionnaire was obtained from the original publisher (Kaneko Shobo, Tokyo, Japan).

Degree of Independence in Bladder and Bowel Management

Children with SB responded to the following questions using a 10-cm-long straight line as a visual analogue scale (VAS): “How much of your bladder management is controlled by yourself?” and “How much of your bowel management is controlled by yourself?” In addition, the parents responded to the following questions: “How much of your child’s bladder management is controlled by him/herself?” and “How much of your child’s bowel management is controlled by the child him/herself?” Both parents and children with SB responded, and the closer the VAS was to 10 cm, the greater the degree of independence.

Analysis

All statistical analyses were conducted using SPSS for Windows (version 27.0; IBM, Tokyo, Japan) with descriptive statistics (mean, standard deviation, median, and interquartile range (IQR)), Spearman’s correlation coefficient, and the Mann-Whitney U test. The correlation between parent and child HRQOL was examined in a group in which the parent and child responses to the VAS were divided by their respective median values and the parent’s and children’s higher or lower VAS group classifications were consistent. Spearman’s correlation coefficients were used to assess the correlation between the QUALAS-C/T-J and SF-8, and the following cutoff values were used: r < 0.20 = very weak; 0.20-0.39 = weak; 0.40-0.59 = moderate; 0.60-0.79 = strong; and 0.80-1.0 = very strong.

## Results

Participants’ characteristics

The questionnaire was distributed to 103 pairs and returned by 93 (response rate: 90.3%), 83 of whom satisfied the inclusion criteria (valid response rate: 89.3%). Table 1 shows the characteristics of the participants. Overall, 48.2% of the children with SB were boys (mean age: 12.91 years), 75.9% of the children with SB were enrolled in a regular school or special class, 89.2% had undergone CIC, and 22.9% had social bladder continence. Mothers were the most common respondents (92.8%). The mean age of the parent respondents was 43.99 years, and 82 couples were married. In addition, 14.5% of the parents had a history of psychiatric visits.

**Table 1:** Participants’ characteristics SB: Spina bifida; CIC: Clean intermittent catheterization

	N (%) or Mean (SD)
Children with SB	
Sex, n (%)	
Male	40 (48.2%)
Female	43 (51.8%)
Age, years	12.91 (2.78)
Type of school, n (%)	
Regular school	41 (49.4%)
Special class	22 (26.5%)
School for special needs education	17 (20.5%)
Mobility, n (%)	
Community ambulator	55 (66.3%)
Household ambulator	3 (3.6%)
Nonfunctional ambulator	5 (6.0%)
Non-ambulator	20 (24.1%)
Ventriculoperitoneal shunt, n (%)	
Yes	74 (89.2%)
No	8 (9.6%)
Unknown	1 (1.3%)
CIC, n (%)	
Yes	74 (89.2%)
No	8 (9.6%)
Unknown	1 (1.2%)
Social bladder continence, n (%)	
Yes	19 (22.9%)
No	59 (71.1%)
Unknown	5 (6.0%)
Bowel continence, n (%)	
Yes	45 (54.2%)
No	37 (44.6%)
Unknown	0 (0.0%)
Respondents with a child with SB	
Respondent’s relationship to child, n (%)	
Father	5 (6.0%)
Mother	77 (92.8%)
Parents	0 (0.0%)
Others (foster mother)	1 (1.2%)
Parent age, years	43.99 (4.97)
Marital status, n (%)	
Married	82 (98.8%)
Divorced/bereaved	1 (1.2%)
Employment status, n (%)	
Full-time	13 (15.7%)
Part-time	38 (45.8%)
Childcare leave	1 (1.2%)
Housewife	24 (28.9%)
Others	7 (8.4%)
Income satisfaction, n (%)	
Extremely dissatisfied	1 (1.2%)
Dissatisfied	10 (12.0%)
Slightly dissatisfied	17 (20.5%)
Neither	28 (33.7%)
Slightly satisfied	20 (24.1%)
Satisfied	5 (6.0%)
Extremely satisfied	2 (2.4%)
Educational level, n (%)	
Undergraduate	26 (31.3%)
Junior/senior high school	18 (21.7%)
Junior college	21 (25.3%)
Special training college	12 (14.5%)
Postgraduate	6 (7.2%)
History of treatment for mental illness, n (%)	
No	71 (85.5%)
In the past	12 (14.5%)
Unknown	12 (14.5%)

Distribution of scores for each instrument

**Figure 1:**
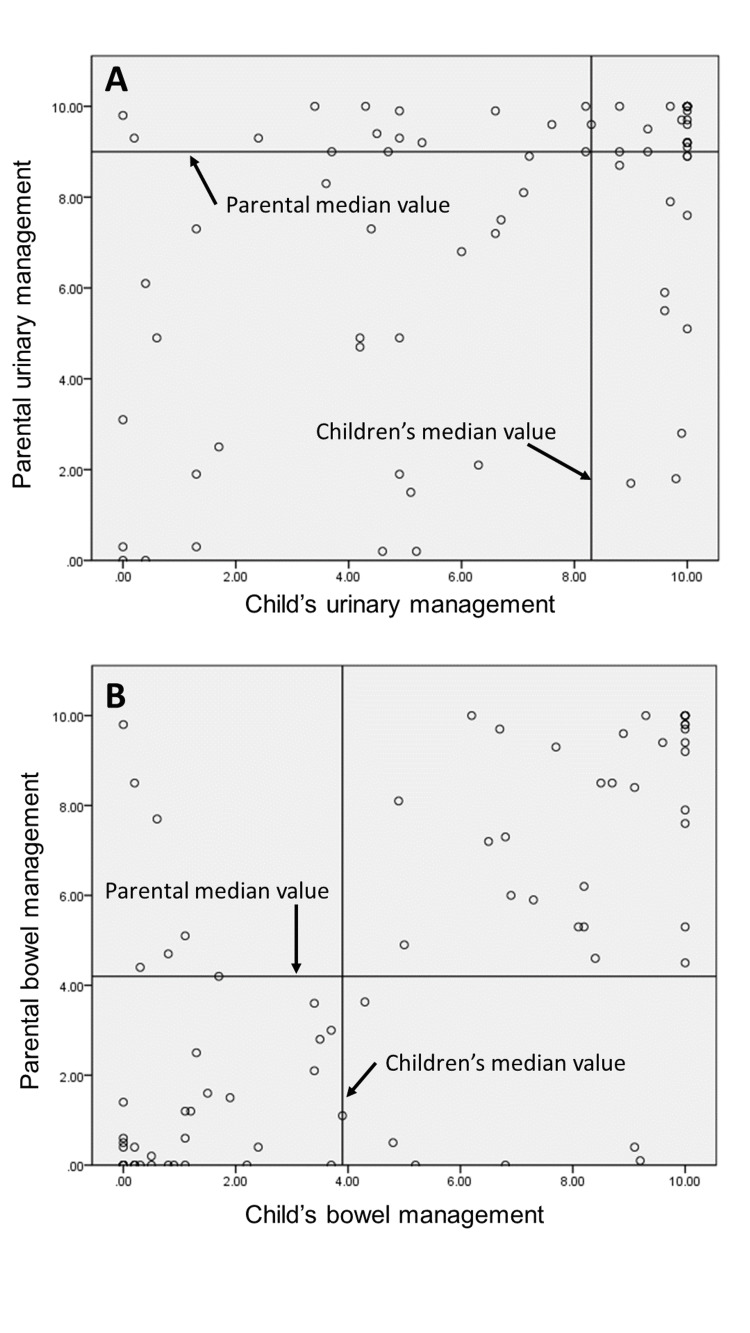
Scatterplot of Bladder and Bowel Control independence for children with SB The figure shows a circle plot of the VAS scores for responses from children with SB and their parents. Panels A and B represent scatterplots of Bladder and Bowel Control independence, respectively. Each median value is shown as a line on the figure. SB: Spina bifida; VAS: Visual analogue scale

Table 2 shows the distribution of scores for each instrument. The total scores and scores of each domain for all instruments were non-parametrically distributed. The median (IQR) values for the Psychosocial and Bladder and Bowel domains of the QUALAS-C/T-J were 80.00 (60.00-95.00) and 70.00 (55.00-85.00), respectively, while the median (IQR) values for the PCS and MCS of the SF-8 were 50.92 (45.95-54.36) and 48.75 (42.58-52.65), respectively. The median (IQR) values regarding the bladder management independence level were 9.00 (5.40-9.90) for parents and 8.30 (4.45-10.00) for children with SB, and the median (IQR) values regarding the bowel management independence level were 3.92 (0.18-8.50) for parents and 4.10 (0.58-9.10) for children with SB. Dividing the VAS scores into high and low groups by each median value, parents and children were classified into the same group: 25 and 31 pairs in the lower and higher independence groups, respectively, for bladder management, and 34 and 35 pairs in the lower and higher independence groups, respectively, for bowel management (Figure 1 and Table 3).

**Table 2:** Distributions of scores for the instruments used in the present study QUALAS-C/T-J: Japanese version of the quality of life assessment in spina bifida for children/teenagers; SF-8: Short Form-8; VAS: Visual analogue scale; PCS: Physical component summary; MCS: Mental component summary; IQR: Interquartile range

	Mean (SD)	Median (IQR)
QUALAS-C/T-J		
Independence	75.35 (21.89)	80.00 (60.00–95.00)
Bladder and Bowel	67.95 (20.59)	70.00 (55.00–85.00)
SF-8		
PCS	49.71 (6.90)	50.92 (45.95–54.36)
MCS	47.09 (7.24)	48.75 (42.58–52.65)
Parent VAS		
Bladder management	7.41 (3.24)	9.00 (5.40–9.90)
Bowel management	4.29 (3.94)	3.92 (0.18–8.50)
Child VAS		
Bladder management (n = 81)	6.88 (3.45)	8.30 (4.45–10.00)
Bowel management (n = 82)	4.74 (3.99)	4.10 (0.58–9.10)

**Table 3:** Parent-child VAS scores for independence levels Parent and child VAS scores were divided by their respective median scores. VAS: Visual analogue scale

			Children’s VAS	
			Lower	Higher
Bladder management independence level	Parental VAS	Lower	25	12
		Higher	15	31
Bowel management independence level	Parental VAS	Lower	34	7
		Higher	7	35

Correlation between parent’s and children’s self-reported HRQOL

Tables 4 and 5 present the results of the Mann-Whitney U test comparing the HRQOL of children with SB and their parents in two groups based on the level of independence in bladder or bowel self-management of the responding children and parents, respectively. No significant differences in the HRQOL domains of the parents and children were observed based on a comparison of the two groups.

**Table 4:** Comparison of HRQOL among children with SB between low and high levels of independence in bladder/bowel self-management Regarding the children’s level of independence (VAS) for bladder and bowel self-management as reported by the children with SB, the two groups were divided into lower and higher levels of independence groups by the median level of independence. The table shows the results of a comparison of children’s HRQOL between low and high levels of independence, and of Hypothesis 1. In the two independence groups, Mann-Whitney U tests were conducted for each domain of the QUALAS-C/T-J. HRQOL: Health-related quality of life; QUALAS-C/T-J: Japanese version of the quality of life assessment in spina bifida for children/teenagers; VAS: visual analogue scale; SB: Spina bifida

Group	n	QUALAS-C/T-J							
		Psychosocial				Bladder and Bowel			
		Mean rank	U	z	p	Mean rank	U	z	p
Bladder self-management level (answered by children with SB)									
Lower	40	40.76	810.50	–0.45	0.65	40.58	803.00	–0.52	0.60
Higher	43	43.15				43.33			
Bowel self-management level (answered by children with SB)									
Lower	41	38.68	725.00	–1.25	0.21	38.93	735.00	–1.15	0.25
Higher	42	45.24				45.00			

**Table 5:** Comparison of HRQOL of parents of children with SB between low and high levels of children’s independence in bladder/bowel self-management Regarding the children’s level of independence (VAS) for bladder and bowel self-management as reported by parents, the two groups were divided into lower and higher levels of independence groups by the median level of independence. The table shows the results of a comparison of parent’s HRQOL between low and high levels of children’s independence, and of Hypothesis 2. In the two independence groups, Mann-Whitney U tests were conducted for each domain of the SF-8 HRQOL scales. HRQOL: Health-related quality of life; SF-8: Short Form-8; PCS: Physical component summary; MCS: Mental component summary; VAS: Visual analogue scale

Group	n	SF-8							
		PCS				MCS			
		Mean rank	U	z	p	Mean rank	U	z	p
Bladder self-management level (answered by parents)									
Lower	37	42.62	828.00	–0.21	0.83	40.28	787.00	–0.58	0.56
Higher	46	41.50				43.38			
Bowel self-management level (answered by parents)									
Lower	41	40.02	780.00	–0.74	0.46	39.54	760.00	–0.92	0.36
Higher	42	43.93				44.40			

Table 6 shows the correlations for each domain of the QUALAS-C/T-J and SF-8. In the total HRQOL analysis, the PCS of the SF-8 was significantly correlated (weakly or moderately) with the Psychosocial and the Bladder and Bowel domains of the QUALAS-C/T-J. Next, a stratified analysis was carried out for responses that were in agreement with parent and child perceptions of independence in bladder and bowel management (Table 3). Regarding the bladder management independence level, tendencies for stronger correlations between the Psychosocial domain of the QUALAS-C/T-J and MCS (weakly vs. very weakly) of the SF-8 were observed in the lower than in the higher group. Meanwhile, the lower bowel management independence level groups showed significantly stronger correlations between the Psychosocial and Bladder and Bowel domains of the QUALAS-C/T-J and PCS of the SF-8 in the lower than in the higher group (strongly vs. weakly; moderately vs. very weakly).

**Table 6:** Total and independence levels of bladder/bowel management—correlation for each QUALAS-C/T-J and SF-8 domain Correlations between the PCS and MCS of the SF-8 and the Psychosocial and Bladder and Bowel domains of the QUALAS-C/T-J are shown. In addition to overall Bladder and Bowel management, each domain of the QUALAS-C/T-J was compared between two groups according to the degree of independence in Bladder and Bowel management. QUALAS-C/T-J: Japanese version of the quality of life assessment in spina bifida for children/teenagers; SF-8: Short Form-8; PCS: Physical component summary; MCS: Mental component summary; VAS: Visual analogue scale ** p < 0.001

Total/stratify	Total		Bladder management				Bowel management			
QUALAS-C/T-J	Psychosocial	Bladder and Bowel	Psychosocial		Bladder and Bowel		Psychosocial		Bladder and Bowel	
Self-management independence level (VAS)			Lower	Higher	Lower	Higher	Lower	Higher	Lower	Higher
SF-8										
PCS	0.41**	0.35**	0.38	0.3	0.18	0.26	0.65**	0.21	0.46**	0.13
MCS	0.05	0.03	0.34	–0.06	0.14	–0.09	0.00	–0.05	–0.10	–0.03
n	n = 83		n = 25	n = 31	n = 25	n = 31	n = 34	n = 35	n = 34	n = 35

## Discussion

In the present study, approximately 90% of the survey participants reported that they had undergone CIC, which is consistent with the 90% advocated in the Clinical Guidelines for Lower Urinary Tract Dysfunction in Patients with Spina Bifida [25]. The finding that about 90% of the children were enrolled in regular classes did not differ from previous studies [21,22]. Thus, the population in the present study appears to be representative of children and teenagers with SB in Japan. Compared with the median level of independence in bladder and bowel management, participants were characterized as having achieved bladder management independence and as being in the process of achieving independence in bowel management.

No significant relationship was observed between parents’ or children’s HRQOL and the children’s degree of bladder and bowel self-management; therefore, Hypotheses 1 and 2 were rejected. Previous studies have reported that SB is a complex disorder associated with various factors, which makes it difficult to establish statistical significance [26]. Therefore, a simple structure linking the degree of independence of children with SB in managing their bladder and bowels to the HRQOL of parents and children may not be observable.

In addition, the strengths of the correlations of HRQOL between all parent and child pairs were weak or moderate in the PCS of the SF-8 and the QUALAS-C/T-J (Hypothesis 3). In a two-group comparison focusing on independence in bladder and bowel management among children with SB, the strengths of the correlations between QUALAS-C/T-J and parents’ HRQOL for children with SB in the lower independence group were strong (PCS and Psychosocial, Bowel management), moderate (PCS and Bladder and Bowel, Bowel management), and weak (MCS and Psychosocial, Bladder management), and stronger correlations were seen compared with the whole analysis (Hypothesis 4). Therefore, the correlation between parent and child HRQOL was stronger than that in the overall analysis, and the correlation between parent and child HRQOL was weaker in the higher independence group. In particular, the strength of the correlation with PCS found in the low independence group for bowel management was comparable to the strength of the correlation between HRQOL for children with asthma and caregivers whose medical adherence depends on caregiver visitation behavior [27]. Previous studies of parents of children with chronic illnesses in the Netherlands, Taiwan, and Turkey have reported that caregivers’ HRQOL is significantly lower in physical than in psychosocial aspects [28-30]. A study in Egypt found that parents are distressed as a result of parental physical restraint in regard to their child’s medical care and medical visitation behaviors [31]. In the present study, in regard to parents and children with lower levels of independence in bowel management, parents might be restrained physically by the child’s bowel management. This may indicate that parents’ physical HRQOL is strongly correlated with their children’s HRQOL. The results of the present study may indicate that interventions for the management of bladder and bowel in children with lower levels of independence in bladder and bowel management have a synergistic effect on the HRQOL of not only the child, but also the parents.

This study has several limitations. First, the study participants only comprised about 40% of the 255 eligible participants. These participants may have been a group with a high interest in SB and a high parent HRQOL. However, it is possible that there were further difficulties in bladder and bowel management in individuals who did not intend to participate or in children with SB and parents who were not members of the patient group, and they may have additional difficulties in bladder and bowel management; therefore, it is necessary to investigate such populations in the future. Next, the QUALAS-C/T-J, the HRQOL instrument for children used in this study, evaluates perceptions of the impact of managing bladder and bowel dysfunction on daily life. On the other hand, the SF-8, the parent’s HRQOL instrument, evaluates the parent’s physical and psychological health status, but cannot assess the perception of how the health status affects their daily lives. In this respect, it is not capable of measuring exactly the same aspects of HRQOL for parents as the QUALAS-C/T-J for children. Therefore, perception-based instruments should be a priority for the measurement of HRQOL in future research. In addition, it is important to investigate the impact of increased independence in bladder and bowel management on HRQOL among children and the perceived impact of children’s bladder and bowel management assistance on their physical and psychological health. Finally, because the present study targeted all children with SB aged 8-17 years, not only those who were in the process of becoming independent in bladder management, this population had a higher degree of independence in bladder management. Therefore, the number of samples comprising the consensus group was small. There were also limitations in terms of capturing individuals in the transition phase of bladder management. Thus, future studies should focus separately on the transition phases of bladder and bowel self-management.

## Conclusions

The results of the present study revealed a significantly stronger correlation between the QUALAS-C/T-J and SF-8 PCS in the lower than in the higher independence group for bowel management. In conclusion, nursing interventions that focus on parent-child interactions specific to the transitional phase could not only improve the HRQOL of children with SB, but also be supported more effectively by the parent-child interaction, thereby enhancing the HRQOL of parents as well.
